# 5-Carboxylcytosine levels are elevated in human breast cancers and gliomas

**DOI:** 10.1186/s13148-015-0117-x

**Published:** 2015-08-21

**Authors:** Maria Eleftheriou, Ana Jimenez Pascual, Lee M. Wheldon, Christina Perry, Abdulkadir Abakir, Arvind Arora, Andrew D. Johnson, Dorothee T. Auer, Ian O. Ellis, Srinivasan Madhusudan, Alexey Ruzov

**Affiliations:** Division of Cancer and Stem Cells, School of Medicine, Centre for Biomolecular Sciences, University of Nottingham, University Park, Nottingham, NG7 2RD UK; Medical Molecular Sciences, Centre for Biomolecular Sciences, University of Nottingham, University Park, Nottingham, NG7 2RD UK; Department of Oncology, Nottingham University Hospitals, Nottingham, NG5 1PB UK; School of Life Sciences, University of Nottingham, University Park, Nottingham, NG7 2RD UK; Department of Academic Radiology, Queen’s Medical Centre, Nottingham University Hospitals, University of Nottingham, Nottingham, NG7 2UH UK; Department of Pathology, Division of Cancer and Stem Cells, School of Medicine, University of Nottingham, Nottingham, NG51PB UK; Academic Unit of Oncology, Division of Cancer and Stem Cells, School of Medicine, University of Nottingham, Nottingham, NG51PB UK; Present address: Lab de Neurophysiologie, Université libre de Bruxelles, Campus Erasme CP 601, Bldg. C Room C3-143, 808, Route de Lennik, B-1070 Brussels, Belgium

**Keywords:** DNA methylation, 5-hydroxymethylcytosine, 5-carboxylcytosine, Carcinogenesis, Breast cancer, Immunohistochemistry

## Abstract

**Background:**

DNA methylation (5-methylcytosine (5mC)) patterns are often altered in cancers. Ten-eleven translocation (Tet) proteins oxidise 5mC to 5-hydroxymethylcytosine (5hmC), 5-formylcytosine (5fC) and 5-carboxylcytosine (5caC). In addition to their presumptive specific biological roles, these oxidised forms of 5mC may serve as intermediates in demethylation process. According to several reports, 5hmC levels are strongly decreased in cancers; however, the distribution of 5fC and 5caC in malignant tissue has not been studied.

**Findings:**

Here, we examine the levels of 5hmC and 5caC in 28 samples of normal breast tissue, 59 samples of invasive human breast cancer and 74 samples of gliomas using immunochemistry. In agreement with previous reports, we show that 71 % of normal breast samples exhibit strong 5hmC signal, compared with only 18 % of breast cancer samples with equivalent levels of 5hmC staining. Unexpectedly, although 5caC is not detectable in normal breast tissue, 27 % of breast cancer samples exhibit significant staining for this modification (*p* < 0.001). Surprisingly, the presence of immunochemically detectable 5caC is not associated with the intensity of 5hmC signal in breast cancer tissue. In gliomas, we show that 5caC is detectable in 45 % of tumours.

**Conclusions:**

We demonstrate that, unlike 5hmC, the levels of 5caC are elevated in a proportion of breast cancers and gliomas. Our results reveal another level of complexity to the cancer epigenome, suggesting that active demethylation and/or 5caC-dependent transcriptional regulation are pre-activated in some tumours and may contribute to their pathogenesis. Larger studies to evaluate the clinicopathological significance of 5caC in cancers are warranted.

## Findings

### Background

DNA methylation (5-methylcytosine (5mC)) is an epigenetic modification, which contributes to the regulation of gene expression in a wide range of biological contexts [1]. The patterns of DNA methylation are altered in a range of cancers [1, 2]. Moreover, hypermethylation of promoters of tumour-suppressor genes has been identified as one of the causes of cancer progression [3].

Ten-eleven translocation (Tet) proteins (Tet1/2/3) contain a C-terminal catalytic dioxygenase domain that oxidises 5mC to 5-hydroxymethylcytosine (5hmC) and further to 5-formylcytosine (5fC) and 5-carboxylcytosine (5caC) [4–6]. A number of recent studies suggest that 5hmC plays a specific role in both transcriptional regulation and DNA demethylation in different biological settings [7, 8]. Importantly, 5hmC is strongly depleted in human cancers [9, 10] and its loss is accompanied with malignant cellular transformation [11, 12].

5hmC has recently attracted considerable attention from cancer biologists; however, the distribution of the two other oxidised forms of 5mC (5fC and 5caC) in malignant tissue has not been studied. Nevertheless, both these marks can be recognised and excised from DNA by thymine-DNA glycosylase (TDG) followed by regeneration of non-modified cytosine via base-excision repair (BER) pathway leading to active Tet/TDG-dependent demethylation of certain genomic regions [5, 6, 13]. Since demethylation of promoters of tumour-suppressor genes has been implicated in tumorigenesis [3], elucidating potential roles of these modifications in cancers would be important for understanding the mechanisms of cancer progression.

We previously showed that although both 5fC and 5caC are immunochemically detectable in mammalian post-implantation embryos with similar patterns of spatial distribution, the intensity of 5fC signal is always considerably weaker than that of 5caC likely due to a lower sensitivity of the 5fC antibody [14]. Thus, in this study, we focused our analysis on 5caC aiming to determine the global levels of this mark in human breast cancers and gliomas.

### Methods

#### Patients and tissue samples

Paraffin-embedded formaldehyde-fixed tissue microarray blocks were used for 5hmC and 5caC immunochemical detection in breast cancers. The microarray blocks contained 97 cores with 59 samples of invasive breast cancer tissues with the rest of the cores representing normal breast tissue. The breast cancer tissue samples were invasive ductal carcinomas from patients who underwent surgery for early stage breast cancers. All tumours were reviewed by expert pathologist at the Nottingham University Hospitals. All tumours were derived from Caucasian patients diagnosed with breast cancer lacking any known germ-line mutations in BRCA1 or BRCA2. The ‘normal’ samples were tumour-associated normal breast tissue removed at the time of definite surgery in the Caucasian patients diagnosed with breast cancer lacking any known germ-line mutations in BRCA1 or BRCA2. Ethical approval for the breast cancer study was granted by the Nottingham Research Ethics Committee 2 under the title ‘Development of a molecular genetic classification of breast cancer’ (C202313). Examination of 5caC distribution in gliomas was performed using 76 paraffin-embedded tumour sections from patients treated at Nottingham University Hospitals between the years 2005 and 2011. Of the glioma patients, 45/74 (60.8 %) were male. The median age of patients at inclusion in the study was 47 years (range 22–81 years). Twenty-seven percent (20/74) of the patients were diagnosed with a low grade glioma (all grade 2). Of the 54 high grade gliomas included in the study, 15/54 (27.8 %) were grade 3 tumours and 39/54 (72.2 %) were grade 4 tumours. In patients with low grade glioma, 12/20 (60.0 %) were male and the median age at inclusion in the study was 33.5 years (range 25–57 years). The median age at study inclusion was 54.5 years (range 22–81 years), and 33/54 (61.1 %) patients with high grade glioma were male. All the samples have been examined by an expert neuropathologist. All the patients were referred from Nottingham, Leicester, Lincoln and Derby, Leicestershire, Northamptonshire and Rutland Research Ethics committee. This study has been approved by the Regional Ethics Committee (reference number 08/H0406/102). Biomax US tissue microarray (BN1002a) was used for fluorescent immunochemistry of normal breast tissue.

#### Immunohistochemistry, microscopy, scoring and statistical analysis

3,3′-Diaminobenzidine (DAB)-based immunohistochemistry was performed using the Novolink kit polymer detection system (Cat. no.RE7280-K, Leica) following the manufacturer’s instructions. The slides were de-waxed and rehydrated according to the standard procedures and incubated in the antigen retrieval citrate solution heated to close to boiling for 20 min. Anti-5hmC (cat. no. 39791; Active Motif, 1:2000 dilution) and anti-5caC (cat. no. 61225; Active Motif, 1:1000 dilution) rabbit polyclonal antibodies were diluted in Leica diluent (cat. no. AR9352) and incubated with the slides for 1 h at room temperature. After incubation with primary antibodies, the slides were washed in TBS and incubated in post primary block solution for 30 min, followed by washes in TBS and incubation with the polymer solution for 5 min. The signal was visualised by incubation of the slides in 1:20 diluted DAB solution for 5 min. Haematoxylin was used for nuclei visualisation. 5hmC signal was evaluated as undetectable (0), weak (1), moderate (2) or strong (3). 5caC staining intensity was assessed as negative (0) or positive (1). Paraffin-embedded formaldehyde-fixed 12.5 dpc murine embryonic tissue was used as a positive control for 5hmC/5caC immunostaining. Images were obtained using a Nikon eclipse 80i microscope. Immunofluorescence immunohistochemistry and confocal microscopy was performed as described in [14]. Control staining without primary antibody produced no detectable signal. Statistical significance of the differences between the distributions of 5hmC and 5caC was determined by the ANOVA single factor test. Chi-squared tests were used to examine associations between two categorical variables.

### Results

**Fig. 1:**
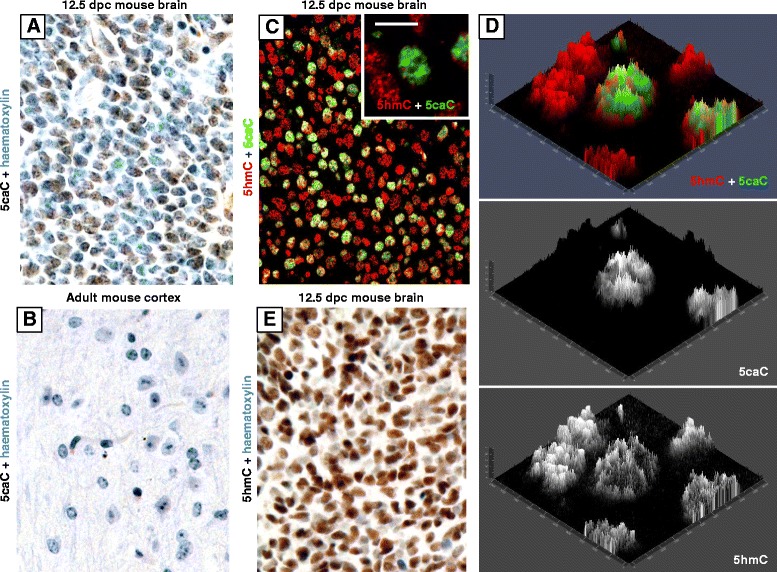
Both DAB-based and fluorescent immunohistochemistry protocols are suitable for 5caC detection in paraffin-embedded tissues. DAB-based detection of 5caC in 12.5 dpc mouse embryonic brain tissue (**a**) and adult mouse cortex (**b**). **c-d** Confocal image of 12.5 dpc mouse embryonic brain tissue immunostained for 5hmC and 5caC using tyramide-based fluorescent immunochemistry. The *inset* shows the magnified field used for the generation of 2.5XD signal intensity plots presented in (**c**). Merged views only are shown in (**c**) and the merged view together with individual channels in (**e**). Scale bar is 10 μm. **e** DAB-based detection of 5hmC in 12.5 dpc mouse embryonic brain tissue

**Fig. 2:**
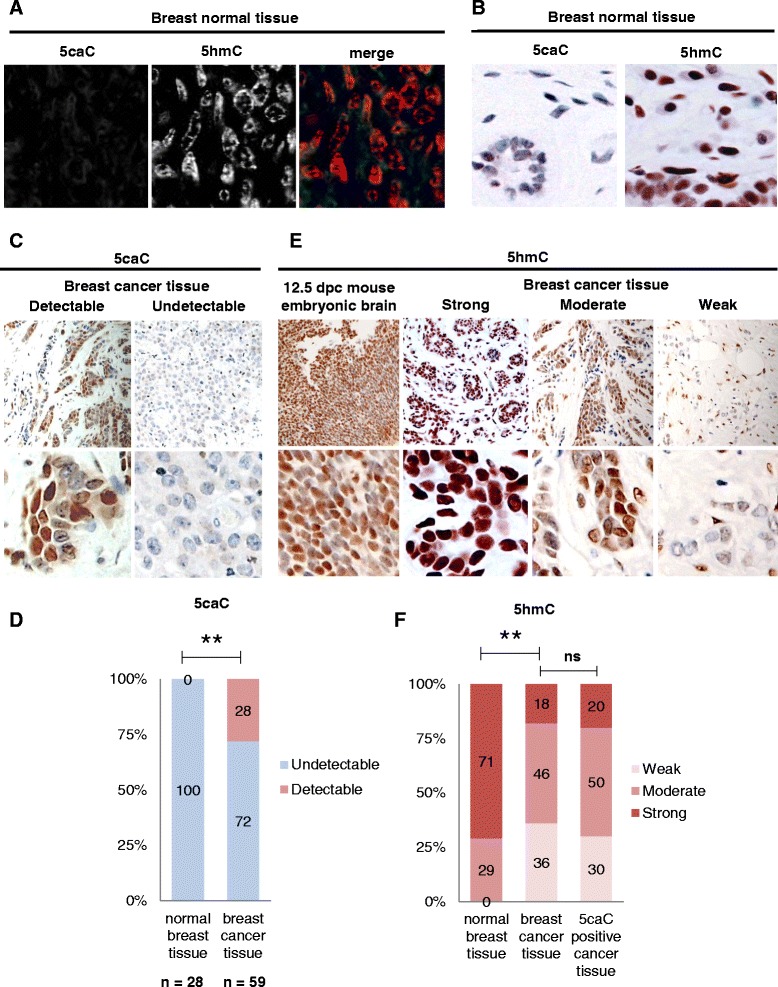
5caC levels are elevated in a range of human breast cancers. **a** Fluorescent 5caC and 5hmC immunostaining in normal breast tissue. Individual channels and merged view are shown. **b** DAB-based 5caC and 5hmC immunostaining in normal breast tissue. **c** Examples of breast cancer tissue with different levels of 5caC signal (designated as detectable or undetectable) used for the categorization of 5caC staining presented in (**d**). **d** Proportions of breast cancers and samples of normal breast tissue with detectable 5caC staining. **e** Examples of breast cancer tissue with different levels of 5hmC signal (designated as strong, moderate or weak) used for the categorization of 5hmC staining presented in (**f**). 5hmC staining in 12.5 dpc embryonic brain is shown as a positive control. **f** Proportions of breast tumours, 5caC positive breast tumours and samples of normal breast tissue exhibiting different levels of 5hmC staining. **p < 0.001

We have previously extensively used fluorescent immunohistochemistry for detection of both 5hmC and 5caC in mammalian and amphibian tissues [14–16]. Moreover, our protocol for immunochemical detection of 5caC was validated by mass spectrometric identification of the modified cytosine bases [14]. To compare DAB-based and fluorescence-based protocols for 5hmC/5caC immunostaining, we initially performed detection of 5hmC and 5caC in mouse embryonic and adult brain tissues using the two immunostaining techniques (Fig. 1a–e). In agreement with our previous results [14], 5caC was detectable in 12.5 days post coitum (dpc) but not in the adult mouse brain by both methods (Fig. 1a–c). Furthermore, whereas 5hmC staining was fairly uniform in 12.5 dpc mouse brain, both DAB-based and fluorescent immunochemistry produced identical characteristic patterns of 5caC distribution in embryonic brain tissue, with staining intensity varying in the cells differentiating towards different lineages [15] (Fig. 1a, c–d). In addition, we obtained identical results from 5hmC and 5caC immunostaining of normal human breast tissue using both versions of immunochemistry. Thus, whereas 5hmC staining of these samples was relatively high, 5caC was immunochemically undetectable in breast tissue samples (Fig. 2a, b).

Since we obtained identical results using both staining techniques, we decided to employ DAB-based immunochemistry for 5hmC/5caC detection in breast cancers. Initially, we examined the levels of 5caC in 59 samples of invasive human breast cancers and 28 samples of normal breast tissue. Although we could not detect any 5caC staining in normal breast tissue samples, a significant number of cancer samples (28 %, *p* < 0.001) exhibited evident 5caC signal (Figs. 2c, d). In contrast, and in agreement with previous studies [9, 10], 71 % of normal breast samples exhibited strong 5hmC signal, whereas only 18 % of breast cancer samples had comparable levels of 5hmC staining. Thus, 5hmC staining was very weak in 36 % and significantly decreased in 46 % of cancer samples (Fig. 2e, f). Surprisingly, the presence of immunochemically detectable 5caC was not associated with the levels of 5hmC immunostaining in the corresponding samples of malignant breast tissue (Fig. 2f).

**Fig. 3:**
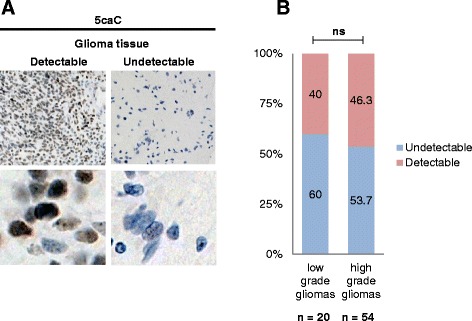
5caC immunostaining in human gliomas. **a** Examples of glioma tissue samples with different levels of 5caC signal (designated as detectable or undetectable) used for the categorization of 5caC staining presented in (**b**). **b** Proportions of low and high grade gliomas exhibiting immunochemically detectable levels of 5caC

To extend our study with another type of cancer, we performed immunochemical detection of 5caC in 74 samples of glioma tissue. Analogous to breast cancers, this modification was detectable in 40 % of low and 46.3 % of high grade gliomas (Fig. 3a, b). Notably, the presence of 5caC was not associated with glioma grade (Fig. 3b).

### Discussion

In one of the first studies reporting decreased levels of 5hmC in malignant tissue, it has been noted that the depleted 5hmC content did not correlate with the levels of Tet1/2/3 expression in a number of tumours [10]. Correspondingly, here, we show that the levels of 5caC are elevated in a considerable fraction of breast cancers we analysed, including the tumour samples producing low intensity of 5hmC staining. This suggests that, at least in some cancers, depleted 5hmC may not necessarily indicate low degrees of Tet-dependent 5mC oxidation. In contrast, it is likely that Tet1/2/3 proteins are pre-activated in certain cancer-related settings leading to the preferential oxidation of 5mC to 5fC/5caC instead of 5hmC. In this context, the presence of high 5caC levels in cancer tissue may point at intensified ratios of active demethylation or, alternatively, at repression of components of BER machinery implicated into removal of this mark from DNA [5, 6, 13]. Thus, both active remodelling of DNA methylation patterns and 5caC-dependent transcriptional regulation may participate in aberrant nuclear reprogramming associated with pathogenesis of cancers [2] adding another layer of complexity to the epigenetic landscape of this disease.

Since the large body of experimental evidence have previously indicated that 5hmC content is decreased in cancers [9–12], it has been counterintuitive to search for more oxidised forms of 5mC such as 5fC/5caC in cancer-related contexts. With an exception of a recent report, which did not find any significant difference in 5caC content between the cancer patient-derived and healthy control blood samples [17], no attempt has been made to systematically assess the global 5caC levels in tumour tissue to date. Therefore, our observation that 5caC and, potentially, DNA demethylation, is elevated in some tumours represents an important beginning for deciphering the roles of this mark in carcinogenesis and opens an avenue for further 5fC/5caC-related studies in cancer systems. Future investigations on genomic distributions of 5fC/5caC in tumour-derived cell lines and larger studies on different types of cancers are necessary to evaluate the functions and clinicopathological significance of immunochemically detectable 5caC in tumour tissue.
